# IMPACTS OF HANDGRIP ON REVERSING FRAILTY PROGRESSION IN OLDER ADULTS: A TWO-YEAR FOLLOW-UP STUDY

**DOI:** 10.1093/geroni/igae098.4003

**Published:** 2024-12-31

**Authors:** Wan Yun Chou, Su-I Hou

**Affiliations:** University of Central Florida, Orlando, Florida, United States; The University of Central Florida, Orlando, Florida, United States

## Abstract

Frailty is characterized by a significant decline in the functional reserves of multiple organ systems, which increases vulnerability to stressors and elevates the risk of adverse health outcomes. Addressing frailty is becoming crucial for both intervention and policymaking. This prospective cohort study, conducted between 2018 and 2020 at a medical center in Taiwan, aimed to assess the needs of individuals at risk of frailty within the community. The study involved 318 participants aged 65 and older who attended annual health examinations. Frailty was measured using Fried’s frailty scale, cumulative deficits, and physical risk variables, assessed through self-reported questionnaires and objective measures at admission, 6, 12, and 24 months. Based on frailty scores, participants were categorized into low-risk and high-risk groups, with 0 indicating low risk and 1 or higher indicating high risk. The study analyzed differences between these groups concerning the five criteria of Fried’s frailty scale: weight loss, low handgrip strength, exhaustion, slow walking speed, and physical inactivity. Wilcoxon signed-rank tests and Generalized Estimating Equation (GEE) models were employed for analysis. 60% (n=193) of participants were considered high-risk. Notably, the decline in grip strength was significantly greater in the high-to-low-risk group compared to the low-to-high-risk group at 12 and 24 months (p=0.036 and 0.016). GEE models revealed a borderline significant reduction in frailty risk associated with improvements in cognitive function (aOR=0.69, 95% CI=0.46-1.04). These findings suggest that enhancing grip strength and cognitive function can contribute to reversing frailty, highlighting the benefits of targeted interventions for older adults.

